# Bioreactor Study Employing Bacteria with Enhanced Activity toward Cyanobacterial Toxins Microcystins

**DOI:** 10.3390/toxins6082379

**Published:** 2014-08-13

**Authors:** Dariusz Dziga, Magdalena Lisznianska, Benedykt Wladyka

**Affiliations:** 1 Department of Plant Physiology and Development, Faculty of Biochemistry, Biophysics and Biotechnology, Jagiellonian University, Gronostajowa 7, 30387 Kraków, Poland; E-Mail: magdalena.sworzen@uj.edu.pl; 2 Department of Analytical Biochemistry, Jagiellonian University, Gronostajowa 7, 30387 Kraków, Poland; E-Mail: wladykab@interia.pl; 3 Malopolska Centre of Biotechnology, Jagiellonian University, Gronostajowa 7, 30387 Kraków, Poland

**Keywords:** microcystin, microbial degradation, bioreactor

## Abstract

An important aim of white (grey) biotechnology is bioremediation, where microbes are employed to remove unwanted chemicals. Microcystins (MCs) and other cyanobacterial toxins are not industrial or agricultural pollutants; however, their occurrence as a consequence of human activity and water reservoir eutrophication is regarded as anthropogenic. Microbial degradation of microcystins is suggested as an alternative to chemical and physical methods of their elimination. This paper describes a possible technique of the practical application of the biodegradation process. The idea relies on the utilization of bacteria with a significantly enhanced MC-degradation ability (in comparison with wild strains). The cells of an *Escherichia coli* laboratory strain expressing microcystinase (MlrA) responsible for the detoxification of MCs were immobilized in alginate beads. The degradation potency of the tested bioreactors was monitored by HPLC detection of linear microcystin LR (MC-LR) as the MlrA degradation product. An open system based on a column filled with alginate-entrapped cells was shown to operate more efficiently than a closed system (alginate beads shaken in a glass container). The maximal degradation rate calculated per one liter of carrier was 219.9 µg h^−1^ of degraded MC-LR. A comparison of the efficiency of the described system with other biological and chemo-physical proposals suggests that this new idea presents several advantages and is worth investigating in future studies.

## 1. Introduction

Microcystins (MCs), hepatotoxic cyclic heptapeptides, produced by several cyanobacterial species, may be transformed into non-toxic derivatives in several different ways in the natural environment. These methods include physical processes, like thermal or light-mediated decomposition, chemical oxidation or enzymatic degradation [1,2,3]. Furthermore, adsorption to suspended or sedimented particles and accumulation followed by transformation in aquatic organisms may occur and impact the concentration of these toxins [4,5,6]. However, these processes are relatively slow and do not play a crucial role in the regulation of MC concentration during and after bloom formation. Within the last few years, a notable amount of new information regarding the biodegradation of cyanotoxins has been published, and several bacteria with degradation activity against MCs were identified in the environmental samples [7]. Such discoveries should provide backing for future research to enable a general understanding of this phenomenon. Another approach is to study the possible practical application of microorganisms capable of cyanotoxin degradation. Recently, several proposals of MC biodegradation processes employed in the removal of cyanotoxins have been published. Among them, biologically-active filters (granular activated carbon and sand) have been suggested as the most attractive water treatment option [8,9,10]. On the other hand, the use of bacteria capable of MC degradation directly in water reservoirs was described by Ji *et al.* [11] and Sumino *et al.* [12] as an alternative to drinking water treatment. In these proposals, the introduction of bacterial strains directly into water reservoirs are suggested, and this is another option that may help in the reduction of MC occurrence below the postulated guideline value. However, such environmental manipulation should be based on local strains capable of MC degradation and requires the preparation of a large volume of bacterial culture.

**Figure 1:**
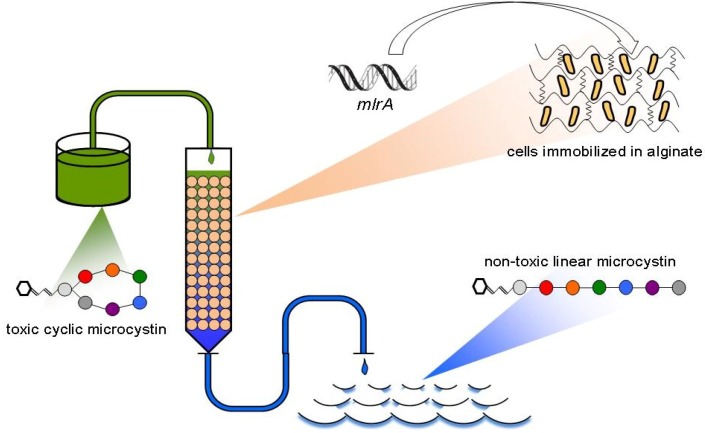
A bioreactor employing BL21(DE3) cells modified according to *mlrA* gene expression and immobilized in alginate beads.

This paper presents a novel proposal based on the utilization of genetically engineered microorganisms (Figure 1). The previously investigated bioreactors employing natural microorganisms offer relatively low efficiency compared to chemical treatment, and their use in fast purification of large amounts of MC-contaminated water is questionable. Our preliminary experiments [13] indicated that bacterial cells with significantly increased biodegradation potency are still active toward MC after immobilization in alginate. In the present paper, several novel data have been provided, including: (1) the comparison of open and closed bioreactors; (2) optimizing the efficiency of MC degradation using a different length of column and different initial microcystin LR (MC-LR) concentration; and (3) assays of the long-term efficiency of columns packed with alginate bead-based MC removal at different temperatures.

## 2. Results

Preliminary experiments for this work were performed to confirm the hypothesis that only a part of the MlrA enzyme produced by BL21 cells is involved in MC degradation. Firstly, *E. coli* BL21-*mlrA* cells with and without induction by IPTG exhibited a similar level of activity (17.4 and 23.7 mU mL^−1^ of cell culture at stationary phase, respectively). This activity was relatively stable during long-term incubation in LB medium 100-fold diluted with freshwater. For this reason, the cultivation of BL21-*mlrA* in the further experiments described below was performed without IPTG induction. Secondly, the study on the location of MlrA in the BL21 cells (cultivated with and without IPTG) indicated that a significant amount of the enzyme is located mainly in cytosol (89.8% and 87.7% of total activity, respectively). In the periplasmic fraction, 5.5% and 5.7% of MlrA activity was noticed, whereas in membranes, 4.5% and 6.8% (induced and non-induced cells, respectively).

**Figure 2:**
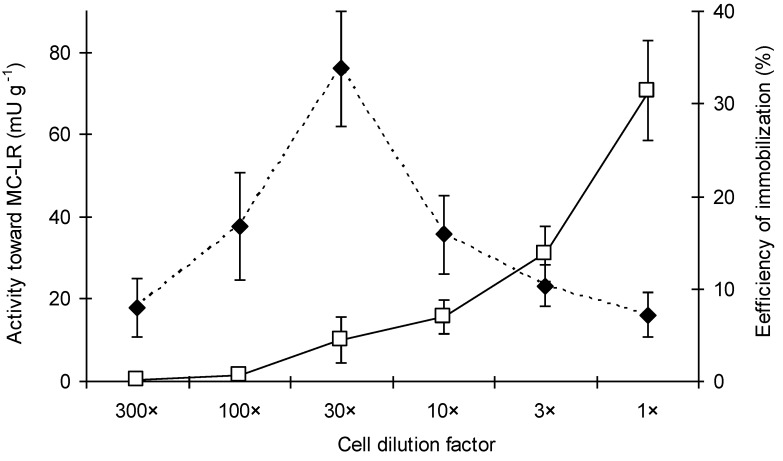
The activity of alginate-entrapped BL21-*mlrA* cells against MC-LR depending on cell concentration. The initial density (1 fold dilution) was 1.2 × 10^10^ cells mL^−1^. The solid line indicates total measured activity of alginate beads, the dashed line indicates calculated efficiency of immobilization. Errors bars indicate standard deviation (*n* = 4).

Different dilutions of cells were tested to optimize the maximal activity of the formed alginate beads with entrapped BL21-*mlrA* cells. The greatest potency of beads for MC degradation was found for the highest investigated cell concentration (Figure 2). On the other hand, the efficiency of immobilization (expressed as the activity of beads towards MC-LR) calculated per dilution fold was the highest for cell dilution factor 30×, and then, it decreased when the density of the culture was enhanced.

The rate and dynamics of degradation were shown to be different in a closed system when compared to beads packed in a column under continuous flow (Table 1). Independently of the column volume and applied concentration of toxin, the fast reduction of MC-LR concentration in the first minutes of the experiment was observed in a glass container with the vortexed beads, but the rate of degradation decreased during the experiment. On the other hand, in an open system, the concentration of MC-LR in the flow, as well as the rate of degradation were stable during the experiment. For example, in an open system filled with 2.23 mL of carrier (3 cm of column length), 49% of MC-LR was transformed into linear form with 1.8 min empty bed contact time (EBCT), and the rate of degradation ranged between 33.4–35.5 µg h^−1^ L^−1^ during 3 h of continuous flow.

**Figure 3:**
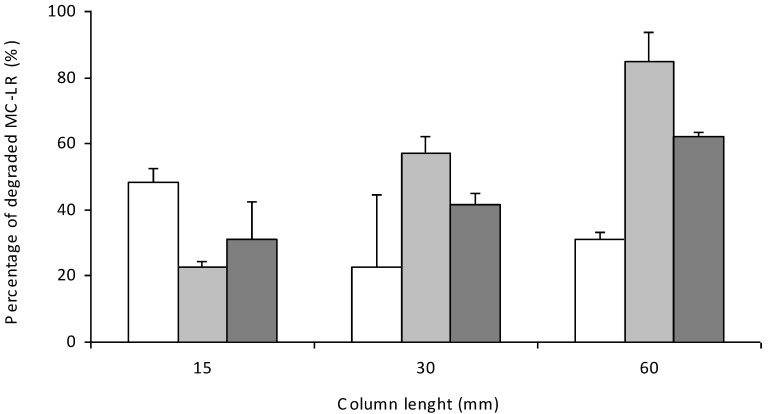
The collective data of MC-LR degradation efficiency depending on the column length and initial MC-LR concentration. White, gray and dark gray blocs are the response to the initial MC-LR concentration (10, 35 and 100 µg L^−1^, respectively). Bars indicate standard deviations (*n* = 9).

**Table 1:** The efficiency of closed and open bioreactors within 3 h of operating in the different initial MC-LR concentration and volume of the carrier.

Heading	Initial MC-LR concentration (µg L^−1^)	Type of system									
		**Batch bioreactor (closed)**					**Column (open)**				
time (min)		5	20	60	120	180	5	20	60	120	180
**volume of carrier 2.23 mL**											
concentration of toxin (µg L^−1^)	**10**	8.77 ± 0.28	8.21 ± 0.43	4.81 ± 0.79	2.26 ± 0.44	1.16 ± 0.61	5.11 ± 0.58	4.94 ± 0.82	4.81 ± 1.07	5.10 ± 0.26	5.06 ± 0.51
total degraded toxin (µg)		0.07	0.11	0.31	0.47	0.76	0.01	0.05	0.15	0.30	0.62
rate of degradation (µg h^−1^ L^−1^)		207.7	78.3	74.5	55.2	31.4	35.5	34.3	33.4	35.4	35.1
**volume of carrier 8.46 mL**											
concentration of toxin (µg L^−1^)	**10**	7.94 ± 0.41	5.31 ± 0.38	1.29 ± 0.20	0.00	0.00	0.92 ± 0.49	1.34 ± 0.08	1.53 ± 0.25	1.29 ± 0.19	1.29 ± 0.40
total degraded toxin (µg)		0.12	0.28	0.52	0.60	0.60	0.02	0.09	0.25	0.52	1.04
rate of degradation (µg h^−1^ L^−1^)		175.1	99.9	61.8	35.5	17.8	32.2	30.8	30.1	30.1	30.9
**volume of carrier 8.46 mL**											
concentration of toxin (µg L^−1^)	**35**	20.64 ± 0.42	7.46 ± 0.38	1.43 ± 0.23	0.00	0.00	5.64 ± 0.62	7.40 ± 0.59	6.99 ± 0.37	4.40 ± 0.16	5.06 ± 0.02
total degraded toxin (µg)		0.25	0.47	0.58	0.60	0.60	0.07	0.28	0.84	1.84	3.31
rate of degradation (µg h^−1^ L^−1^)		349.5	167.6	68.1	35.9	18.0	104.2	98.0	99.4	99.4	108.6

Different lengths of columns and different initial concentrations of MC-LR were tested (Figure 3) to find the optimal efficiency of MC-LR degradation in an open system. The tested MC-LR concentration ranged between 10 and 100 µg L^−1^, which is typical during blooms [14]. As could be expected, the length of the column improved the efficiency. In a six-centimeter-column, 86.5% and 85.0% of MC-LR was degraded within a short time for a concentration of 10 and 35 µg L^−1^, respectively; the estimated EBCT was only 3.6 min. In the case of a much higher concentration of 100 µg L^−1^, which is extremely rare in nature, the efficiency of degradation was 62.3%. The highest documented rate of degradation in open system (calculated per 1 L of carrier) was 219.9 µg h^−1^ of degraded MC-LR (100 µg L^−1^ initial concentration, six-centimeter-column, 0.5 mL min^−1^ flow rate, 20 °C).

**Figure 4:**
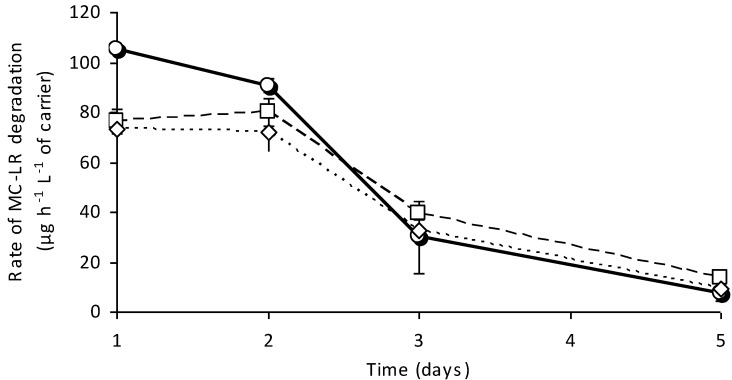
The rate of MC-LR degradation (µg h^−1^ L^−1^ of carrier) in relation to the temperature in a column (6-cm length) packed with alginate-entrapped BL21-*mlrA* cells during five days of continuous flow. The initial MC-LR concentration was 35 µg L^−1^. Solid, dashed and dotted lines show the response to the column temperatures of 20, 15 and 10 °C, respectively. Error bars indicate the standard deviation (*n* = 3).

Previously [13], the activity of cells immobilized in alginate beads was shown to be unstable during long-term incubation (three weeks). In a subsequent experiment, the dependence of MC degradation capability on the temperature and long-term stability of the designed system was analyzed (Figure 4). The columns were under a continuous flow of freshwater (0.5 mL min^−1^) during the experiment, and the MC-LR degradation potency was tested on the first, second, third, fifth and 28th day. The initial efficiency of the columns incubated at 20, 15 and 10 °C was 85.0% ± 3.5%, 61.6% ± 4.0% and 58.8% ± 5.5%, respectively. During two days of continuous flow, the efficiency of degradation was relatively stable, whereas in the following days, it was reduced below 35% (third day) and 12% (fifth day), which may be the result of the decreased viability of the cells.

## 3. Discussion

### 3.1. Biodegradation of MCs as a Promising Alternative

The proposed biotechnological systems for the removal of cyanotoxins from drinking water indicated that biologically-active filters may be an alternative water treatment option [9,15]. Granular activated carbon (GAC) filters can be applied both for the adsorption and biodegradation, whereas the removal of MCs in sand filters has been shown to be primarily through biological degradation processes. Other materials have also been applied in the biological filtration of cyanotoxins, such as glass beads, porous ceramic materials and plastic media. So far, such proposals have assumed the use of naturally occurring bacterial strains immobilized on different carriers. However, bioreactors that employ wild strains still seem to act relatively slowly in comparison with the chemo-physical treatment of water. A possible solution to this problem may be a significant increase of the MC degradation potency of the used microorganisms. The idea of the present research is based on the utilization of genetically engineered microorganisms (GEMs).

Despite the potential risks of impacting the environment due to the persistence of undesired genes and their transfer to indigenous species, the application of GEM has been proposed as an attractive method in biological wastewater treatment and bioremediation of soil or groundwater. *Pseudomonas fluorescens* HK44, which is able to degrade polyaromatic hydrocarbons, is the first genetically engineered bacterium that has been approved by the U.S. Environmental Protection Agency for use in bioremediation of soils in the field [16]. Recently, several genetically engineered microorganisms have been successfully constructed for bioremediation purposes [17,18,19,20]; however, environmental concerns and regulatory constraints limit their application *in situ*. In this work, the construction and usage of genetically modified bacteria is assumed; however, the action of such bacteria is limited to the space of bioreactors, and no release into the environment is expected.

Within the last two years, some manuscripts documenting the heterologous expression of Mlr proteins have been published. Such an approach provides several advantages, e.g., better biochemical characterization of these important enzymes, as well as verification of their role in microcystin degradation [21,22,23,24]. Such research also provides broader perspectives for future studies and gives crucial background for original application systems for utilizing dangerous water components. The bacteria with enhanced MC-degradation capability require (in practice) the expression of only the MlrA enzyme with access to MCs, because the linear form of MCs are, in environmentally relevant concentrations, non-toxic [21].

### 3.2. MlrA Location

The cells of the *E. coli* BL21-*mlrA* strain tested previously [21] have indicated relatively high activity against MCs, three orders of magnitude higher than the natural degrader, the *Sphingomonas* AMC-3962 strain. Additionally, the same ratio of activity was noticed when cells of both strains were immobilized in alginate [13]. Based on the compared activity of cells and cell extracts (ratio 1:440, [21]), it was suspected that only a small portion of MlrA acts directly in the intact cells. Present data confirm such hypothesis, because an analysis of the MlrA location in BL21 cells indicated that a large portion of MlrA is located in cytosol, whereas only a few percent of MlrA activity was found in the periplasm. The possible explanation of these results may be that only a few percent of the enzyme molecules is secreted outside the inner membrane of bacteria, where the linearization of MC probably occurs. Such results suggest that the transformed BL21 cells do not use the whole potency and that a large portion of the MlrA is not employed in MC decomposition. The construction of other recombinant *mlrA* variants with a sequence delivering the enzyme into the periplasmic space may be a possible way of increasing the efficiency of MC degradation by living cells.

### 3.3. Efficiency of Bioreactors

In our recent report, we indicated that immobilization of MC-degrading bacteria in alginate is a promising option [13]. Among different natural polysaccharide matrices used in the immobilization technique for microorganisms, entrapment in alginate is one of the most commonly employed [25]. To mimic the natural condition, MC-LR solution was always prepared by dilution in freshwater from Dobczyckie Lake. To obtain the maximal activity of alginate beads against MC and the maximal potency of bioreactors, the highest tested value of cell condensation is preferred (Figure 2) for future experiments. However, this strategy requires a larger volume of cell culture and results in a lower immobilization efficiency. It implies a higher production cost of cells with the desired properties. In future experiments, different parameters must be tested to optimize the proportion of cells and alginate and to find more efficient immobilization conditions, as well. The choice between “closed” and “open” systems of water treatment poses another problem. In closed bioreactors, a certain volume of water with MC-LR was treated with alginate beads for different periods of time (which may be controlled), whereas in open systems, the volume of water to be purified was not limited, but the time of treatment was dependent on the flow rate. Our results indicated that the usability of the tested systems depends on the common initial concentration of MCs (Table 2). An open system is more suitable when water with a lower level of toxins (10 µg L^−1^) is purified. In such a condition the MC-LR concentration is reduced immediately to the level close to that recommended by WHO, whereas in bath bioreactor, this value is obtained after 60 min of incubation. In the assay with 35 µg L^−1^ of MC-LR, the closed system seems to be more effective, because the recommended MC level may be reached in 60 min, whereas the column allows only partial purification of water with the MC-LR concentration significantly above the recommended level. On the other hand in both tested concentrations, the degradation rate is stable during the continuous flow of treated water, which is a big advantage in comparison with a batch bioreactor. In a closed system with a mechanical mix, the rate of degradation was very high in the first 5 min, but this parameter drastically decreased over time, and a dependence of the rate of degradation on MC-LR concentration was observed. Intensive mixing increases the probability of contact between MC molecules and the cells; however, when the MC-LR concentration is reduced (in a closed bioreactor), the interaction of MlrA with MC molecules are changed and the degradation rate decreases, as well. In an open system, continuous flow allows regular contact between the contaminant and the cells, thus, the rate of degradation may be stable for a longer period. Based on these results, an open system was analyzed in the subsequent study, as it is more suitable for continuous biodegradation of MCs. In the experiments with different column lengths and different MC-LR concentrations (tested in an open system, Figure 3), both parameters influenced the frequency of contact between MC-LR and the alginate-entrapped cells. At the initial concentration of MC-LR 10 µg L^−1^, the concentration of toxin leaking from the column was close to the WHO recommendation (1.4 µg L^−1^). The system should be scaled-up in the future from laboratory to technical size, but we assume that in a longer column and at a relevantly faster flow rate, a similar value of MC-LR degradation efficiency may be expected.

**Table 2:** Compared results of selected research on different MC removal systems.

Cited work	Type of reactor	Initial MC concentration (µg L^−1^)	Calculated parameters of MC removal	
			Rate of degradation (µg h^−1^ L^−1^)	Efficiency (%)
[26]	Slow sand filter with *Sphingomonas* MJ-PV strain inoculated in column	50	1.3	80
[27]	Cells of B-9 strain immobilized on polyester pieces in closed container	200	7.5	90
[8]	Morgan WTP filter sand packed in column colonized by bacteria with *mlrA* gene	20	40	100
[28]	Photocatalytic degradation in continuous treatment system	5	225.0 ^a^	85
present work	Column filled with alginate beads, BL21(DE3)-*mlrA* cells immobilized in gel	10	30.5	86
		35	105.5	85
		100	219.9	62
		35	3.8 ^b^	−

Notes: ^a^ Rate of degradation of the whole system consisting of four 2.6 L reactors lit with four mercury vapor lamps (125 W) and treated with TiO_2_ (250 mg L^−1^); ^b^ rate of MC-LR degradation after four weeks of continuous flow.

### 3.4. Stability of Designed System

A crucial parameter of bioreactors is their stability in the natural environment. Our system does not provide stable operation at high intensity for a long period, and the rate of degradation decreases over time (Figure 4). This means that to obtain better efficiency, the flow rate must be relevantly reduced for longer column operation. However, even after significant efficiency decrease, the rate of MC-LR degradation observed in a bioreactor operated at 20 °C after four weeks of continuous flow is still comparable to bioreactors that employ the natural strains (Table 2).

The calculated rate of degradation documented Bourne *et al.* [26] and Tsuji *et al.* [27] was several times lower than in our system. Only the work of Ho *et al.* [8] indicated a relatively fast degradation of MC (up to 20 µg L^−1^) in 7.5 min of empty bed contact time (Table 2). The initial rate of degradation documented in our work is also comparable with the recently published system, which assumes photocatalytic degradation of MCs [28], (Table 2). The previous proposals assumed that the bioreactors operate for several weeks or months and are able to regenerate, which is possible because the cells can proliferate inside the carrier. Additionally, the competition ability of the used natural strains is essential. However, in the cited works, the acclimation phase is required, which makes fast preparation of the system problematic. The proposal presented in this work is different. The tested stability of the system is short (a few days) and seems to be only slightly dependent on the temperature (Figure 4). However, the initial MC-LR degradation rate is very high and, after a few weeks, still similar to bioreactors proposed earlier [26,27]. This experiment indicates that despite its low stability, the proposed system may nevertheless operate better in comparison with some of the previous solutions. The application of alginate-entrapped modified cells may be different. Such a bioreactor can provide a quick reaction in sudden risk, in case of a fast bloom formation or a massive release of MCs from the cells; the documented rate of degradation depends on the initial MC concentration and is faster when the level of MCs is high. In such a situation, a high degradation rate is crucial and more important than stability. Such a column filled with carrier, which is extremely active toward MCs, may be employed in the purification of fish ponds or water reservoirs for the irrigation of fields.

## 4. Experimental Section

### 4.1. Materials

Trifluoroacetic acid (TFA) and sodium alginate were from Sigma (St. Louis, MO, USA). The RP C18 Purospher column was obtained from Merck (Darmstadt, Germany). MC-LR was extracted from a culture of Microcystis aeruginosa PCC 7813 strain obtained from the Pasteur Institute (Paris, France) and HPLC purified, as described earlier [29]. Escherichia coli BL21(DE3) (Novagen, an Affiliate of Merck KGaA, Darmstadt, Germany) with pET21a-*mlrA* used for the expression of recombinant proteins was grown at 37 °C in LB broth supplemented with ampicillin (100 µg mL^−1^).

### 4.2. Expression of Recombinant MlrA

The pET21a-*mlrA* construct obtained using a procedure described earlier [21] was transformed into *E. coli* BL21(DE3), and the bacteria were plated on LB agar plates supplemented with ampicillin (100 µg mL^−1^). Before the experiments described below, a fresh culture was prepared by incubation in LB medium supplemented with ampicillin for 24 h, until the absorbance at 600 nm (A_600_) was in the range 1.5–2.0. In the experiments that required the induction of recombinant expression, the temperature was decreased to 30 °C when A_600_ = 0.8 was reached (approximately after 4 h) and IPTG (isopropyl β-D-thiogalactoside) at a final concentration of 1 mM was added; then, culturing was continued for 20 h. Subsequently, the bacteria were centrifuged (15,000× *g*, 10 min, 4 °C), and the pellet was further processed, as described below.

### 4.3. Location of MlrA Activity in Cellular Fractions

IPTG-induced and non-induced cells of 24 h-old *E. coli* BL21(DE3) cultures were centrifuged for 15 min (6,000 rpm) and washed with 100 mL of 50 mM Tris-NaCl buffer, pH 7.5. All subsequent steps were performed at 4 °C. The periplasmic fraction was isolated by suspension of pellet (1:2, *w*/*v*) in 100 mM Tris buffer (pH 7.5) with 750 mM sucrose and 1 mg mL^−1^ of lysozyme. Cells were gently shaken for 1 h. Then, EDTA was added (1 mM final concentration), and the incubation was continued for 45 min. After the addition of 5 mM MgCl_2_ (final concentration), the pellet was centrifuged for 20 min at 6,000 rpm to obtain the periplasmic fraction. The cytosolic fraction was prepared by sonication of the pellet in 50 mM Tris buffer, pH 7.5 containing 300 mM NaCl, 1 mM PMSF and 1 mM EDTA. Subsequently, the centrifuged pellet (used to isolate the membrane fraction) was suspended in the same buffer containing 1% (*v*/*m*) of DDM (n-dodecyl β-D-maltopyranoside) and gently shaken overnight, then centrifuged at 30,000 rpm for 30 min. The MlrA activity assays were performed for all of the fractions according to the procedure described previously [21].

### 4.4. Immobilization of E. coli BL21-mlrA on Alginate

The immobilization procedure was performed as described previously [13]. Forty milliliters of freshly cultured bacteria of *E. coli* BL21-*mlrA* (18 h-old culture, OD_600_ ≈ 2.0) were centrifuged and resuspended in 2 mL of 50 mM phosphate buffer, pH 7.0. The cell suspension was mixed with 60 mg of slowly added sodium alginate. Next, it was dropped into 5% CaCl_2_ to obtain beads of approximately 1 mm in diameter, which were then incubated in 5% CaCl_2_ for half an hour at 5 °C. The MlrA activity of the intact *E. coli* BL21-*mlrA* cells was measured as described earlier using the HPLC method [13]. The rate of MC degradation was calculated by monitoring the level of linear MC-LR.

### 4.5. Activity of Alginate Beads with Entrapped E. coli BL21-mlrA

The dependence of activity of alginate beads toward MC-LR on the cells concentration was documented by the measurement of MC-LR degradation in glass vials. The beads were prepared according to the procedure described above (Section 4.4*.*); however, different dilutions of the cells were used (3-, 10-, 30-, 100-, 300-fold dilution; the initial density was 1.2 × 10^10^ cells mL^−1^). Ten beads (0.45 g of the total mass) were placed in the glass vials filled with 1 mL of MC-LR solution (0.5 µg mL^−1^). The level of linear MC-LR was monitored after 5, 20, 60 and 120 min of incubation at 20 °C by HPLC (20 µL injection volume). MlrA activity was expressed in mU (one unit is defined as the amount of MlrA that catalyzes the production of 1 µM of linear MC-LR per minute).

### 4.6. Degradation of MC-LR in the Bioreactors

In a closed bioreactor, a glass container was incubated at 20 °C and filled with 60 mL of MC-LR dissolved in filtered freshwater from Dobczyckie Lake. Alginate beads with entrapped cells were mixed on a magnetic stirrer. The open system of water treatment (Figure 1) consisted of an Econo Gradient Pump (Bio-Rad, Hercules, CA, USA), a 13.5-mm diameter glass column filled with the beads, which formed different amounts of the carrier, and a Retriever 500 autosampler (Isco, Lincoln, NE, USA). The constant flow of the MC-LR solution (10, 35 and 100 µg L^−1^) was 0.5 mL min^−1^, and the column was thermostated in Jetstream 2 plus column thermostat (Waters Corporation, Milford, MA, USA). In the both systems (closed and open), the level of linear MC-LR was measured after 5, 20, 40, 60, 120 and 180 min. The procedure was repeated 3 times using new, freshly prepared beads. In the 5-day experiment, three columns incubated at different temperatures (20, 15 and 10 °C) were used simultaneously.

### 4.7. HPLC Assays

HPLC analyses, including the MC-LR degradation rate and identification of the products, were performed using a Waters HPLC system (Waters Corporation, Milford, MA, USA) consisting of a 600E multisolvent-delivery system, a 717 plus autosampler, a 996 photodiode array detector (PDA), Millenium32 SS software and a Jetstream 2 plus column thermostat. MC-LR and its degradation product were quantified on a Purospher STAR RP-18 endcapped column, 55 mm × 4 mm, 3 µm particles (Merck, Darmstadt, Germany), as described by Meriluoto and Spoof [30]. The mobile phase consisted of a gradient of 0.05% aqueous TFA (Solvent A) and 0.05% TFA in acetonitrile (Solvent B) with the following linear gradient program: 0 min 25% B, 5 min 70% B, 6 min 70% B and 6.1 min 25% B.

## 5. Conclusions

The obtained results are important in assessing the benefits and obstacles associated with their applications in the removal of cyanobacterial contaminants. Further research should focus on several important parameters, of which the crucial ones are: (1) construction of bacteria with higher activity toward MCs; (2) finding the best immobilization techniques and the optimization of such a procedure; and (3) the study of the long-term stability of the designed systems in natural conditions. Such knowledge is necessary to design efficient bioreactors for MC utilization.
